# Supplementary material for: Optimization of Insect Odorant Receptor Trafficking and Functional Expression Via Transient Transfection in HEK293 Cells
Source: Chem Senses. 2019 Aug 31;44(9):673–82. doi: 10.1093/chemse/bjz062 (PMC6821309; doi:10.1093/chemse/bjz062)
Supplement: bjz062_suppl_Supplementary_Material [file bjz062_suppl_supplementary_material.pdf]

Optimization of insect odorant receptor trafficking and functional expression via transient transfection in HEK293 cells

Fabio Miazzi, Carolin Hoyer, Silke Sachse, Markus Knaden, Dieter Wicher, Bill S. Hansson, Sofia Lavista-Llanos

Supplementary Information

Primer name	Sequence (5’→3’)
**E.hOr47a_fwd**	GCCCTCTAGAGCCGCCACCATGGATGGAAAACCCATCCCAAATCCTCTTC
**hOr47a_fwd**	GCCCTCTAGAGCCGCCACCATGGATGGAAAACCCATCCCAAATCCTCTTCTGGGGCTTGATAGCACC-GACTCTTTCCTCCAAGTGCAGAAATC
**(E.)hOr47a_rev**	GCCGCTCGAGTCAAGAAAAGCTG
**pCMVTNT_fwd**	CGGTTATCCAGATCGCCCTTCCCAACAG
**pCMVTNT_rev**	CCTGATAAATATAAATGTACATATTATGATATAGATACAACGTATGC
**pBI-CMV1_fwd**	GTACATTTATATTTATCAGGGTTATTGTCTCATG
**pBI-CMV1_rev**	AAGGGCGATCTGGATAACCGTATTACCG
**hOrcoExon1_fwd**	TTAGTGAACCGTCAGATCCGCTAGGGATCCGCCACCATGGAACAGAAACTGATCTCTGAAGAAGACCTG-GCTAGCACAACTAGCATGCAAC
**hOrcoExon1_rev**	CCTTGATACTTACCTGGGCCAGAGCATAGCC
**chimeric_intron_fwd**	TGCTCTGGCCCAGGTAAGTATCAAGGTTACAAGACAGG
**chimeric_intron_rev**	TGCAAAAGTGAAACACCTGTGGAGAGAAAGGCAAAG
**hOrcoExon2_fwd**	TTTCTCTCCACAGGTGTTTCACTTTTGCATTTTC
**hOrcoExon2_rev**	TGATCCTCTGGAGATATCGTCGACAAGCTTTCACTTCAGTTGGACCAG
**BI-R.E.Or47a_fwd**	AACCGTCAGATCGCCTGGAGGCCGCCACCATGGATCAA
**BI-R.E.Or47a_rev**	CCCGCGGCATATGACCGGTGTCAAGAAAAGCTGCGCAGC
**hOr56a_fwd**	GTTTAGTCTGTTCAAGGTAAAGGACTTGTTG
**hOr56a_rev**	CCCGCGGCATATGACCGGTGTCAGTACAGATGAGAACTCCTC
**hOr56aTag_fwd**	AACCGTCAGATCGCCTGGAGGCCACCATGGATCAAGTC
**hOr56aTag_rev**	CTTTACCTTGAACAGACTAAACTTGTTAACGGTG
**pBI-CAG_for**	GGGGTCATTAGTTCATAGCCCATAT
**pBI-CAG_rev**	CAAAACCGCATCACCATGGTAATAG
**pBI-Orco_for**	GTGTACGGTGGGAGGTCTATATAAG
**pBI-Orco_rev**	GTGGTATGGCTGATTATGATCCTCT
**pBI-OrX_for**	CATTTTATGTTTCAGGTTCAGGGGG
**pBI-OrX_rev**	GGCCTATATAAGCAGAGCTCGTTT

Supplementary Table 1. Primers used in this study.

Platform	Plugin (Pl) or package (Pk) name	Citation
**ImageJ**	Image Stabilizer (Pl)	K. Li, "The image stabilizer plugin for ImageJ," http://www.cs.cmu.edu/~kangli/code/Image_Stabilizer.html, February, 2008.
**R**		R core team (2017). R: A language and environment for statistical computing. R Foundation for Statistical Computing, Vienna, Austria. URL http://www.R-project.org/.
**R**	ggplot2 (Pk)	H. Wickham. ggplot2: Elegant Graphics for Data Analysis (Wickham, 2016)
**R**	gridExtra (Pk)	(Auguie, 2017) gridExtra: Miscellaneous Functions for "Grid" Graphics. R package version 2.3. https://CRAN.R-project.org/package=gridExtra
**R**	ggthemes (Pk)	(Arnold, 2017) ggthemes: Extra Themes, Scales and Geoms for 'ggplot2'. R package version 3.4.0. https://CRAN.R-project.org/package=ggthemes
**R**	scales (Pk)	(Wickham, 2017) scales: Scale Functions for Visualization. R package version 0.5.0. https://CRAN.R-project.org/package=scales
**R**	multcomp (Pk)	Torsten Hothorn, Frank Bretz and Peter Westfall (2008). Simultaneous Inference in General Parametric Models. (Hothorn et al., 2008)
**R**	extrafont (Pk)	(Chang, 2014) extrafont: Tools for using fonts. R package version 0.17. https://CRAN.R-project.org/package=extrafont
**R**	drc (Pk)	Ritz, C., Baty, F., Streibig, J. C., Gerhard, D. (2015) Dose-Response Analysis Using R (Ritz et al., 2015)
**R**	DescTools	Andri Signorell. 2019. DescTools: Tools for Descriptive Statistics. Version 0.99.28 https://cran.r-project.org/web/packages/DescTools/index.html
**R**	FSA (Pk)	Ogle, D.H. 2017. FSA: Fisheries Stock Analysis. R package version 0.8.17.
**RStudio**		RStudio Team (2016). RStudio: Integrated Development for R. RStudio, Inc., Boston, MA URL http://www.rstudio.com/.

Supplementary Table 2. Software plugin and packages used for data analysis.

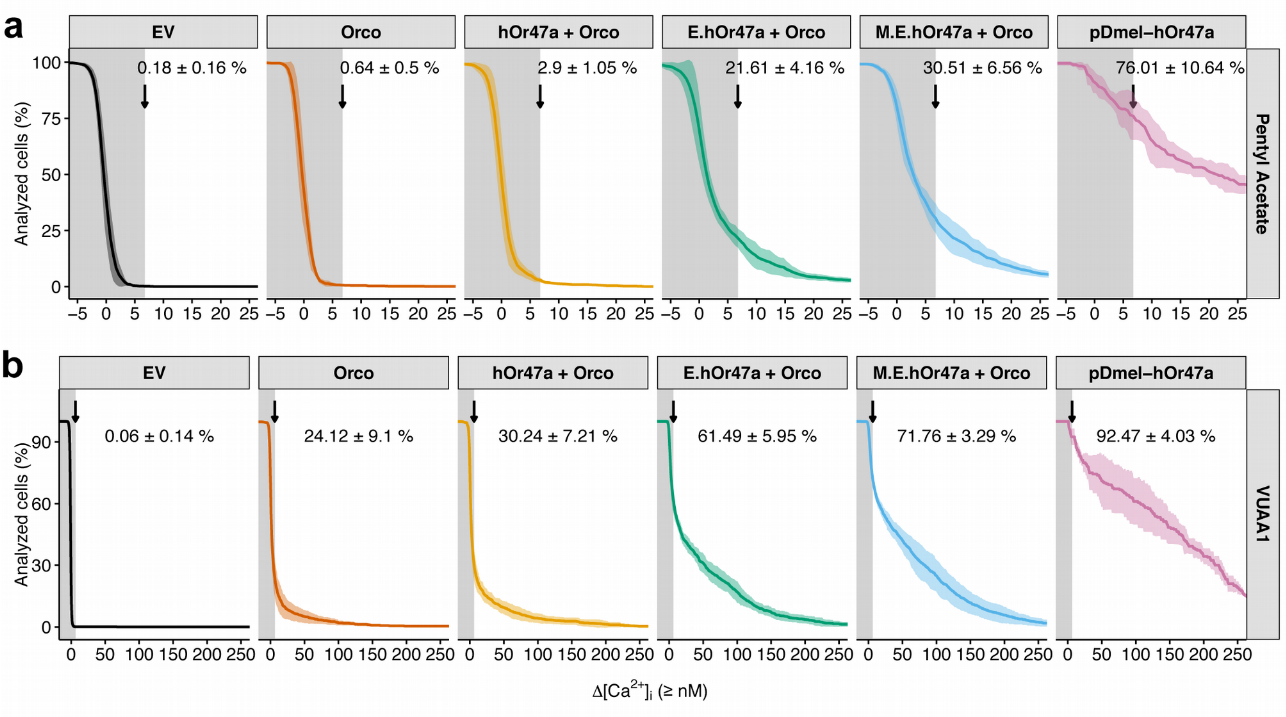

Supplementary Figure 1. Distribution of calcium responses in transfected HEK293 cells

Distribution of the $\Delta[Ca^{2+}]_i$ in transfected HEK293 cells following a stimulation with a 100µl of 100 µM pentyl acetate (a) or 100 µM VUAA1 (b). (a) The $\Delta[Ca^{2+}]_i$ values were calculated for each cell 50 s after stimulation (Time = 100 s in Figure 1c and Figure 2c). Cells were identified as "responding" to the stimulus if a stimulation with pentyl acetate induced a $\Delta[Ca^{2+}]_i \geq 6.75$ nM (non shaded area of the graphs). This threshold value is defined as the mean + 2 × SD response intensity ($\Delta[Ca^{2+}]_i$, in nM) value of the top (most responsive) 0.5 percentile of the cumulative distribution of analyzed cells in the control (Empty Vector) group. (b) The $\Delta[Ca^{2+}]_i$ values were calculated for each cell 20 s after stimulation (Time = 380 s in Figure 1c and Figure 2c). Cells were identified as "responding" to the stimulus if a stimulation with VUAA1 induced a $\Delta[Ca^{2+}]_i \geq 6.25$ nM (non shaded area of the graphs). This threshold was defines in the same was as for (a). The percentage of responding cells is reported for each panel as mean ± SD. Graphs represent mean ± SD. $3 \leq n \leq 5$ for each graph, each distribution (n = 1) is constituted by a number of cells x, with $56 \leq x \leq 355$.

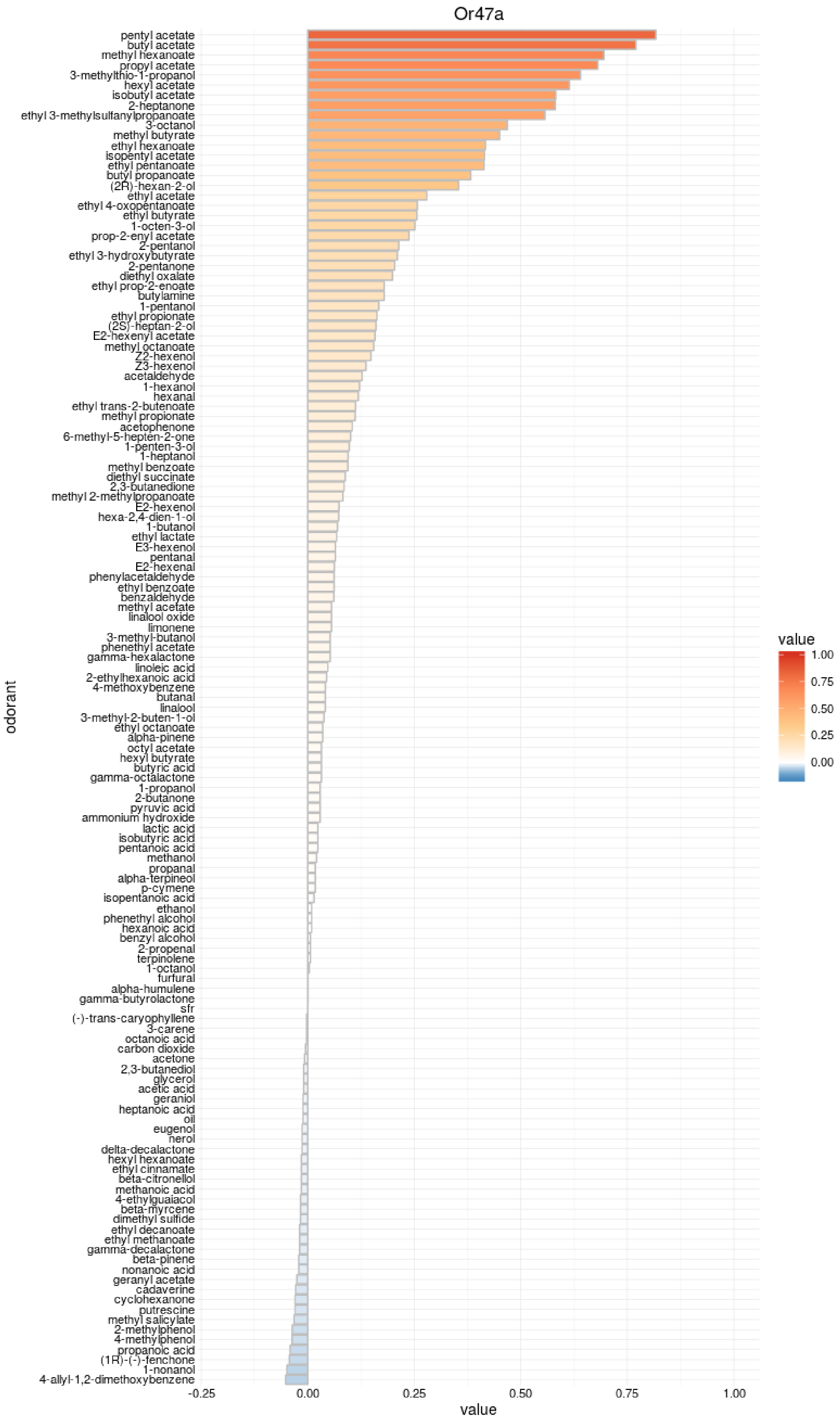
Or47a
odorant
value
pentyl acetate
butyl acetate
methyl hexanoate
propyl acetate
3-methylthio-1-propanol
hexyl acetate
isobutyl acetate
2-heptanone
ethyl 3-methylsulfanylpropanoate
3-octanol
methyl butyrate
ethyl hexanoate
isopentyl acetate
ethyl pentanoate
butyl propanoate
(2R)-hexan-2-ol
ethyl acetate
ethyl 4-oxopentanoate
ethyl butyrate
1-octen-3-ol
prop-2-enyl acetate
2-pentanol
ethyl 3-hydroxybutyrate
2-pentanone
diethyl oxalate
ethyl prop-2-enoate
butylamine
1-pentanol
ethyl propionate
(2S)-heptan-2-ol
E2-hexenyl acetate
methyl octanoate
Z2-hexenol
Z3-hexenol
acetaldehyde
1-hexanol
hexanal
ethyl trans-2-butenoate
methyl propionate
acetophenone
6-methyl-5-hepten-2-one
1-penten-3-ol
1-heptanol
methyl benzoate
diethyl succinate
2,3-butanedione
methyl 2-methylpropanoate
E2-hexenol
hexa-2,4-dien-1-ol
1-butanol
ethyl lactate
E3-hexenol
pentanal
E2-hexenal
phenylacetaldehyde
ethyl benzoate
benzaldehyde
methyl acetate
linalool oxide
limonene
3-methyl-butanol
phenethyl acetate
gamma-hexalactone
linoleic acid
2-ethylhexanoic acid
4-methoxybenzene
butanal
linalool
3-methyl-2-buten-1-ol
ethyl octanoate
alpha-pinene
octyl acetate
hexyl butyrate
butyric acid
gamma-octalactone
1-propanol
2-butanone
pyruvic acid
ammonium hydroxide
lactic acid
isobutyric acid
pentanoic acid
methanol
propanal
alpha-terpineol
p-cymene
isopentanoic acid
ethanol
phenethyl alcohol
hexanoic acid
benzyl alcohol
2-propenal
terpinolene
1-octanol
furfural
alpha-humulene
gamma-butyrolactone
sfr
(-)-trans-caryophyllene
3-carene
octanoic acid
carbon dioxide
acetone
2,3-butanediol
glycerol
acetic acid
geraniol
heptanoic acid
oil
eugenol
nerol
delta-decalactone
hexyl hexanoate
ethyl cinnamate
beta-citronellol
methanoic acid
4-ethylguaiacol
beta-myrcene
dimethyl sulfide
ethyl decanoate
ethyl methanoate
gamma-decalactone
beta-pinene
nonanoic acid
geranyl acetate
cadaverine
cyclohexanone
putrescine
methyl salicylate
2-methylphenol
4-methylphenol
propanoic acid
(1R)-(-)-fenchone
1-nonanol
4-allyl-1,2-dimethoxybenzene
-0.25
0.00
0.25
0.50
0.75
1.00
value
1.00
0.75
0.50
0.25
0.00

Supplementary Figure 2. Expected odor tuning properties of *D. melanogaster* Or47a

Expected response profile for *D. melanogaster* Or47a according to the DoOR 2.0 database. Query retrieved on 24 April 2019 at http://neuro.uni-konstanz.de/DoOR/default.html.

Supplementary Code. ImageJ and R files used for data analysis

Code used in this study is available on GitHub at the following URL: https://github.com/fmiazzi/ORs_HEK293.git

Fura2_ImageJ.js: JavaScript code used to calculate $[Ca^{2+}]_i$ and perform cell segmentation for Figure 1-2.

Figure_1-2.R: R analysis for data shown in Figure 1-2 and Supplementary Figure 1.

Figure3.R: R analysis for data shown in Figure 3.

References

Arnold, J.B. 2017. ggthemes: Extra Themes, Scales and Geoms for "ggplot2".

Auguie, B. 2017. gridExtra: Miscellaneous Functions for "Grid" graphics.

Chang, W. 2014. extrafont: Tools for using fonts.

Hothorn, T., Bretz, F., and Westfall, P. 2008. Simultaneous Inference in General Parametric Models. Biom J. 50:346–363.

Ritz, C., Baty, F., Streibig, J.C., and Gerhard, D. 2015. Dose-Response Analysis Using R. PLOS ONE. 10:e0146021–e0146021.

Wickham, H. 2016. Ggplot2: elegant graphics for data analysis. Springer International Publishing.

Wickham, H. 2017. scales: Scale Functions for Visualization.
